# Reducing radiation exposure in pediatric cervical spine imaging for trauma: a multi-disciplinary quality improvement initiative

**DOI:** 10.1007/s00381-025-06754-z

**Published:** 2025-02-03

**Authors:** Nina Yu, Jonathan Emerson Kohler, Kendra Grether-Jones, Maureen Murphy, Marike Zwienenberg

**Affiliations:** 1 University of California, Davis, School of Medicine, Sacramento, CA 95817 USA; 2 Department of Surgery, University of California, Davis, Sacramento, CA 95817 USA; 3 Department of Emergency Medicine, University of California, Davis, Sacramento, CA 95817 USA; 4 Department of Neurological Surgery, University of California, Davis, 4860 Y St., Suite 3740, Sacramento, CA 95817 USA

**Keywords:** Pediatric, Trauma, Imaging, Quality improvement

## Abstract

**Purpose:**

Pediatric cervical spine injury (PCSI) can result in devastating neurologic disability. While computed tomography (CT) imaging is both sensitive and specific in detecting clinically significant injuries, indiscriminate utilization can lead to excessive ionizing radiation exposure. A routine institutional audit revealed CTs were inappropriately obtained 54% of the time. This study evaluates the effects of an updated protocol to reduce radiation exposure in pediatric trauma patients.

**Methods:**

Data were retrospectively analyzed from a pediatric level 1 trauma center from 2021 to 2022. The data were divided into two cohorts, pre-implementation (2021) and post-implementation (2022). Inclusion criteria were patients 0–14 years old with a Glasgow Coma Scale (GCS) ranging 9–15. Outside-hospital transfers were excluded. The primary study endpoints were guideline compliance and CT utilization.

**Results:**

A total of 82 subjects were enrolled in this study. In 2021, there were 38 subjects (female/male 15/23, mean age 5.9 years old) with an average GCS of 13.6. In 2022, there were 44 subjects (female/male 19/25, mean age 5.2 years old) with an average GCS of 14.0. In 2021, the overall protocol adherence rate was 81.6%, and post-implementation in 2022, compliance was 93.2% (*p* = 0.109). Following implementation, the rate of inappropriate (protocol non-adherent CT) use decreased from 58.6 to 6.8% (*p* < 0.05).

**Conclusions:**

Implementation of a new evidence-based institutional protocol for PCSI was associated with improved adherence and reduction of unnecessary CT orders. Ongoing monitoring will help determine if these improvements are sustained.

## Background

Pediatric cervical spine injury (PCSI) may lead to severe and burdensome neurologic disability; therefore, screening for PSCI leading to prompt diagnosis and treatment is necessary for safe care of injured children. Computed tomography (CT) scans, magnetic resonance imaging (MRI), and X-ray imaging are used in PCSI clearance, while no consensus exists on cervical spine clearance in children [1–4]. As a result, significant variation in the utilization of imaging modalities to achieve clearance exists across centers in the USA. Due to the lack of available dedicated pediatric protocols, adult clearance protocols have been adapted and applied to the pediatric population which may not be appropriate. Children have unique anatomical features which may make them more susceptible to injuries such as Spinal Cord Injury Without Radiographic Abnormality (SCIWORA) [5], which are not seen in the adult population, and which would not be detected if an adult CT-based protocol would be applied to achieve clearance. In addition, there is evidence that using a CT-based approach in children can lead to overexposure to ionizing radiation which is associated with an increased risk of malignancy later in life [6]. For this reason, a more judicious approach in children is warranted.

**Fig. 1:**
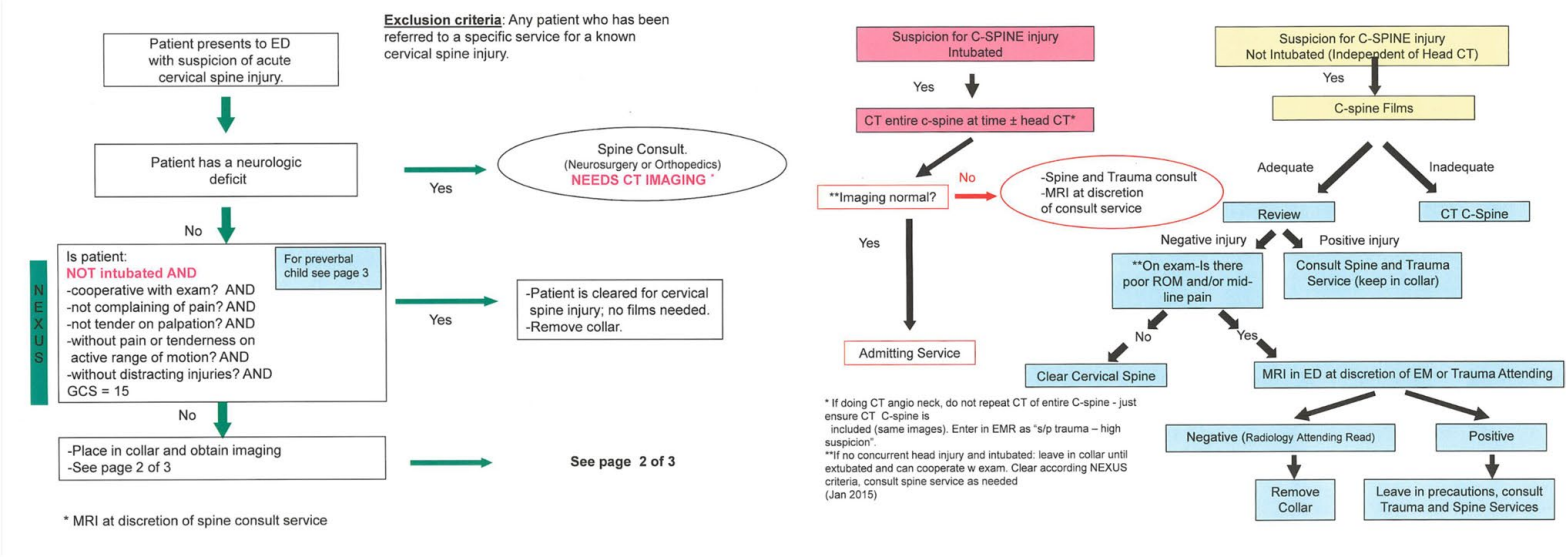
Previous protocol for pediatric cervical spine clearance

Our institution is a Level 1 adult and pediatric trauma center that serves a large population across a wide region. Consensus-based protocols have guided our decision-making for suspected spine injuries with the aim of optimizing the quality of care, patient safety, and value. Protocol adherence in our institution is routinely reviewed as part of the American College of Surgeons (ACS) Trauma Quality Improvement Program. During a 2019 internal review, it was found that CT to clear a suspected PCSI was excessively used in pediatric patients of 8 years old and younger, and the decision to obtain CT imaging was often made by employing our adult institutional guidelines, even though a formal pediatric spine clearance protocol was formulated and shared by the different clinical services involved in pediatric trauma care (Fig. 1). As a result, CT images were appropriately obtained only 46% of the time in the patients included in this audit, and 54% of patients were found to be over-imaged with CT C-spine, including patients who could have been cleared clinically or with support of simple spine X-rays, as formulated in our protocol. In addition, the protocol only included patients up to 8 years of age. With this chosen age cut-off, older children and young adolescents were automatically included in the adult institutional C-spine clearance protocols, which rely much more heavily on obtaining a full CT C-spine to accomplish clearance, further increasing the number of children exposed to increased ionizing radiation.

**Fig. 2:**
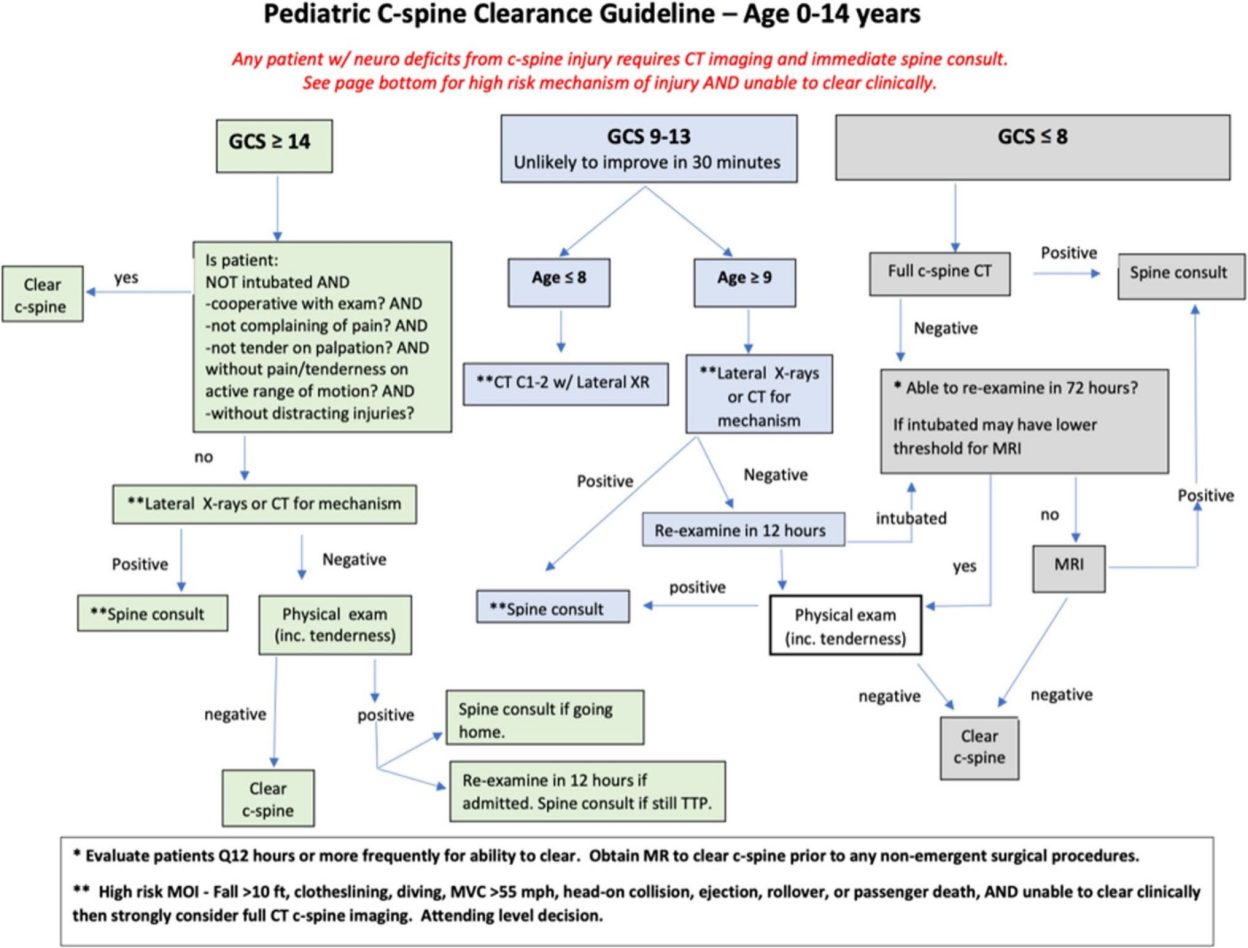
New protocol for pediatric cervical spine clearance. Patients were divided in 3 injury severity groups based upon the presenting GCS. In the low and intermediate injury severity groups, patients were predominantly cleared using clinical examination first and supplementation with lateral spine X-rays and spine consultation if the patient could not be cleared. In the intermediate injury group, patients were re-examined in 12 h if they could not be cleared initially and subsequently triaged to undergo imaging with MRI if they could not be cleared within 72 h of injury. In the high injury severity group, all patients were initially assessed with a full C spine CT and MR was used in those patients not anticipated to be extubated/cooperative for clearance within 72 h

These findings and considerations prompted the formation of a multidisciplinary Pediatric Cervical Spine Work Group (PCSWG) with providers from disciplines including neurological surgery, trauma surgery, pediatric surgery, and emergency medicine, to review and update the pediatric C-spine clearance protocol. The PCSWG sought to develop a new clearance protocol in accordance with the most recent published literature with a focus on reducing unnecessary CTs. In addition, parameters were developed within the protocol for utilization of consultative services and decision-making to improve emergency department throughput and outpatient follow-up as well as formulating imaging recommendations for patients who could not be cleared in the first 24 h after injury. The new C-spine protocol was based on a consensus statement published by Herman et. al. in 2019 [2] and adapted to include these throughput measures as well as more comprehensive inpatient guidelines for MR imaging (Fig. 2). It was approved by the PCSWG and implemented by the departments represented in the group 1 year after the PCSWG formation. In this study, we evaluate our success in adapting a new protocol by comparing protocol adherence before and after implementation.

## Methods

Data were extracted from institutional Trauma and Neurosurgery Traumatic Brain Injury (TBI) databases. Subjects include patients ages 14 years and younger who were seen for TBI with documented cranial injury on the admission CT scan from February 2021 through December 2022. Patients with Glasgow Coma Scale (GCS) of 9–15 were included; patients in whom clearance by clinical exam with or without cervical spine X-rays could be entertained. Patients who were comatose upon admission (GCS 8 and below) automatically underwent cervical spine CT per our institutional protocol. Subjects who were transferred from referring hospitals and those with incomplete records were excluded. The primary study endpoints were guideline compliance and appropriate CT utilization using the updated, consensus-based, C-spine clearance protocol (Fig. 2). A notable imaging adaptation to Herman protocol [2] that the PCSWG agreed upon was to automatically include CT imaging limited to C1-2 with the head CT obtained in children younger than 8 years of age in the moderate TBI group. This adaptation was made to capture odontoid injuries perceived by our group as difficult to assess in young children with routine radiographs. A C-spine CT at our institution involves imaging from the base of the skull (including the craniocervical junction) through the T1. The average effective dose for pediatric C-spine CT examinations was 1.08 mSv at our institution during the period from February 2021 to December 2022. The average effective dose for a pediatric C-spine X-ray is < 0.10 mSv based on published data [7].

For each individual patient, the admission medical record was reviewed, the clinical and imaging data was analyzed, and a determination was made of the expected management according to the protocol active in the period of review. Subsequently, the actual obtained imaging study and steps to clear the C-spine were reviewed in the progress notes to determine the observed management. If expected and observed management were in alignment, the cervical spine clearance process was deemed protocol-compliant. The utilization of full CT scanning of the cervical spine to obtain clearance was analyzed separately and the proportion of patients in whom it was obtained inappropriately, i.e., not in alignment with the cohort protocol, was recorded for each group. Data were exported into Excel and analyzed using chi-square testing with a significance level of 5%.

## Results

A total of 82 subjects were enrolled in our study. In 2021 during the pre-implementation period, there were 15 females and 23 males. The mean age was 5.9 years old. The average GCS was 13.6 with a proportional composition of: 47.4% GCS of 15, 21.1% GCS of 14, 7.9% GCS of 13, 5.3% GCS of 12, 2.6% GCS of 11, 13.2% GCS of 10, and 2.6% GCS of 9 (NS). During the post-implementation period, there were 19 females and 25 males. The mean age of the implementation group was 5.2 years old. The average GCS was 14.0 with a proportional breakdown of the following: 63.6% GCS of 15, 15.9% GCS of 14, 2.3% GCS of 13, 4.5% GCS of 12, 6.8% GCS of 11, 4.5% GCS of 10, and 2.3% GCS of 9 (NS).

In 2021, the overall protocol compliance rate was 81.6%, and post-implementation in 2022, compliance was 93.2% (*p* = 0.1094). In 2021, there were seven overall protocol violations. Of these, full cervical spine CT was used to clear the C-spine in 85.7% of cases. While there was a reduction down to three protocol violations post-implementation, CT was also utilized 100% of the time in these instances.

The historic cohort audited in 2019 included 24 patients, and non-protocol adherent CT was used in 14 patients (58.3%). By comparison, in the pre- and post-implementation cohorts, non-protocol adherent CT imaging was used in respectively 7 out of 38 (18.4%) and 3 out of 44 (6.8%) patients (*p* < 0.05). Following protocol implementation, no patients were found to have a missed cervical spine injury or underwent surgical stabilization for an unstable cervical spine.

## Discussion

The implementation of a new evidence-based institutional protocol for PCSI evaluation demonstrated improved adherence and a reduction in the utilization of unnecessary CTs in children, a potentially significant improvement from our historical CT utilization rate, estimated to be inappropriate or non-protocol compliant in 54% of children. In this study, we observed that immediately preceding the formal implementation of a new cervical spine clearance algorithm, adherence to existing guidelines and protocol compliance increased to 87.5%, and following implementation, it further increased to 93.2%, although not statistically significant.

The historic high rate of protocol violations and excessive use of CT scanning in children to clear the C-spine may have been partly due to our practice environment. At our institution, a level 1 regional pediatric and adult trauma center, children, and adults with trauma are cared for by multidisciplinary teams who treat patients of all ages with most clinical services heavily involved in both adult and pediatric trauma care. As a result, and combined with a lack of standardized pediatric algorithms, adult trauma guidelines—which direct clinicians to use CT scanning to clear the cervical spine in a population of similar injury severity—may more readily be applied in this setting, a practice that has also been reported in the literature [8].

Improved adherence immediately prior to implementation—relative to historical data—may have been due to the Hawthorne effect whereby behavior modification occurred in response to increased scrutiny of clinical management around the time of the PCSI revision. Alternatively, increased collective awareness of the stakeholders during the process of PCSI revision may have influenced behavior and reduced inappropriate CT utilization prior to formal new protocol implementation. These pre-implementation changes are commonly observed in clinical quality improvement processes and may explain the comparatively modest improvement following formal implementation observed in our study. Due to differences in data collection and analysis which predominantly evaluated appropriate CT imaging use rather than protocol adherence before this change in protocol, we could not formally compare adherence in the historical and study cohorts but could compare the frequency of CT scans and protocol-nonadherent CT imaging.

Throughout this protocol revision, various disciplines collaborated to update the PCSI guidelines. While established quality improvement methodologies such as “Lean” or “Six Sigma” were not utilized, the spirit of these methods is shared in our work. The “Lean” principles of quality improvement aim to eliminate wasteful parts of a process to optimize value and the “Six Sigma” model aims to optimize quality and specifically within the healthcare setting, targeting both quality and patient safety [9–11]. These methodologies are often used interchangeably and even more frequently combined as the “Lean Six Sigma” model demonstrated success across multiple parts of the healthcare system [10, 12, 13]. Intrinsic to the Lean Six Sigma model is collaboration across disciplines and personnel to define value and exchange ideas. As multiple disciplines came together to update this protocol, the PSWG was mindful of eliminating any part of the process that could lead to extraneous CT imaging and aimed to optimize patient care. These multidisciplinary meetings focused on stakeholder engagement and were intentional with respect to building a consensus-based protocol. Building an algorithm based on agreement from all stakeholders was a painstaking process that required a lot of time but resulted in two benefits. First, it was an opportunity for those involved in clinical care to participate in the decision-making process and carry ownership over the protocol. Every protocol step was scrutinized and evaluated with questions involving training and availability of support in the event there was uncertainty of how to proceed. Second, frequent reporting was shared to the entire pediatric trauma program which helped expose individuals involved in clinical care to the plans of protocol change and grow support for the new protocol. Improvement in advance of implementation suggests that conversation and widespread awareness of and discussion of the audit results and plans for protocol revisions across multiple contexts and stakeholder groups may hold a significant weight in impacting change.

Another possibility to explain the decreased utilization of imaging pertains to timing. That is, 2021 was marked by the persistence of the COVID-19 pandemic which disrupted society globally. Literature indicates that in the healthcare setting, clinical imaging was used less frequently throughout the pandemic [14, 15], and therefore, more conservative imaging use may also have been practiced at our institution during this time. Other studies of the period of the COVID-19 pandemic have demonstrated similar findings in the pediatric population. In Brazil, imaging of pediatric TBI decreased during the pandemic [16]. Satoskar et al. found that despite the increased incidence of pediatric TBI during the pandemic, CT imaging did not increase concordantly [17].

The literature to date highlights variability not only in terms of protocol details but also in the rates of protocol adherence following implementation. C-spine CT imaging rates before protocol implementation in the pediatric population have a wide range, from 5.4 to 100% [8]. Following protocol implementation, Pennell et al. found that using their standardized protocol in a pediatric cohort aged 18 years and younger caused a 62.8% decrease in CT scans from 2017 to 2018 [4], while Rosati et al. and Connelly et al. found their cohorts to have a decrease of 23% and 25% in CT imaging following implementation of their protocols, from an unspecified 12-month period and 2011 to 2014, respectively [4, 18]. Differences in protocol implementation and adherence seem to predominantly depend upon institutional differences that uniquely prioritized elements such as patient age, utilization and availability of MRI and X-ray, the status of patient consciousness, and NEXUS criteria. For instance, the upper age limit for pediatric protocols ranges from 14 years and younger to 18 years and younger [3, 4, 18]. Additionally, some centers elect to use CT if XR findings were inadequate [1] while other institutions opted for CT only if XR findings indicated a fracture or bony displacement [4]. Mindful of the literature, our healthcare center built upon Herman’s 2019 protocol as it included recommendations from a diverse multidisciplinary group and contained a straightforward and lean algorithm which we believed could improve protocol compliance. Our modifications were based on the addition of secondary steps such as MRI clearance and guidelines to facilitate ED throughput safely. Our institution’s improved protocol adherence rate of 93.2% reflects a 14.2% increase in protocol adherence from baseline and a slightly higher adherence rate than other reports in the literature [3].

The variability in protocols and protocol adherence between institutions highlights the need for larger collaboration across healthcare centers to develop best practices for maximizing protocol adherence. The recently published PECARN study, examining over 22,000 children aged 0 to 17 years, may be helpful in achieving a more unified approach to these patients. Our consensus protocol, although more detailed and specific to an individual patient scenario, falls largely in line with these recommendations, further affirming the role of clinical examination rather than CT imaging in safely achieving cervical spine clearance in the majority of pediatric patients [19].

## Limitations

This retrospective review is a single institution study, subject to limitations inherent to being from one center. That is, the results may not be generalizable to the greater population and observations could be different in other general Trauma or Stand-alone children’s hospitals. Moreover, this study solely focuses on our efforts to implement a new protocol and it does not include a formal assessment of patient outcomes or missed injuries, although no patients in the post-protocol group had a delayed diagnosis of a cervical spine injury or surgical intervention to address a spine injury during hospitalization. Additionally, the relatively small sample size limits the power of our study and ability to detect any differences and therefore limits our ability of making more definitive recommendations. This study also does not measure precise radiation doses from CT scans, though we did not change our CT C-spine radiation dose as part of this protocol change. Minimizing radiation dose when CT scans are indicated is an active area of inquiry in radiology and may be an area of further study at our institution. Lastly, this study focuses on the PCSI protocol for patients with concurrent mild and moderate TBI, GCS 9–15 with documented intracranial or skull injury, and it does not consider patients with isolated cervical spine injuries.

## Conclusion

This study indicates that our updated protocol can be an efficacious means of reducing unnecessary radiation exposure in the pediatric population, potentially reducing the cost burden associated with imaging, and reallocating subspecialty attendings and residents to other areas of patient care needs. Ongoing monitoring will allow us to determine if these improvements are sustained and future multi-institutional studies may help discern the applicability of this protocol across various trauma centers and settings.

## Data Availability

No datasets were generated or analyzed during the current study.
